# KUTE: Green–Kubo Uncertainty-Based Transport Coefficient Estimator

**DOI:** 10.1021/acs.jcim.4c02219

**Published:** 2025-03-19

**Authors:** Martín Otero-Lema, Raúl Lois-Cuns, Miguel A. Boado, Hadrián Montes-Campos, Trinidad Méndez-Morales, Luis M. Varela

**Affiliations:** † Grupo de Nanomateriais, Fotónica e Materia Branda, Departamento de Física de Partículas, 16780 Universidade de Santiago de Compostela, Campus Vida s/n, Santiago de Compostela E-15782, Spain; ‡ Instituto de Materiais (iMATUS), 16780 Universidade de Santiago de Compostela, Avenida do Mestre Mateo 25, Santiago de Compostela E-15782, Spain

## Abstract

An algorithm for the calculation of transport properties from molecular dynamics simulations, kute, is introduced. The method estimates the integrals from the Green–Kubo theorem, taking into account the uncertainties of the correlation functions in order to eliminate arbitrary cutoffs or external parameters whose values could alter the result. In this contribution, the performance of kute is tested against other popular methods for the case of a protic ionic liquid for a variety of transport properties. It is found that kute achieves the same degree of accuracy as the equivalent formulation of the Einstein relations while performing better than other methods to calculate transport properties using Green–Kubo methods.

## Graphical abstract

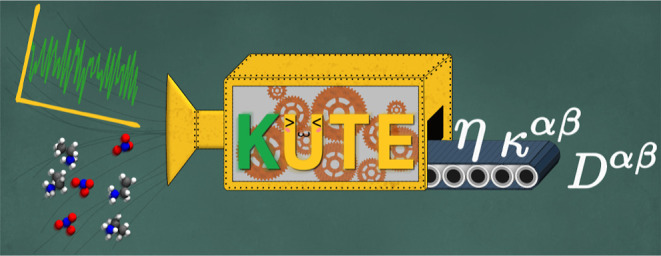

## Introduction

Since the first successful simulations of liquid argon carried out by Rahman^1^ in 1964, molecular dynamics (MD) has established itself as a critical tool for understanding the behavior of matter. Among its most remarkable achievements, we can find its ability to predict the experimental properties of the studied systems with good levels of accuracy, thus enabling the screening of materials for certain applications without the need of synthesizing them.^2–5^ This results in a more efficient search for materials with novel properties, where MD simulations can be used to select the best candidates from a large number of possibilities; something that if done experimentally would incur in high economic costs. Moreover, it is often the case that MD simulations can provide additional insights into the microscopic picture within the material that are difficult to access experimentally.

When it comes to the prediction of experimental characteristics, transport properties are revealed as one of the most relevant metrics to gauge the performance of the materials. These properties relate the macroscopic flow of some measurable quantity; such as heat, electric charge, or momentum, to the gradients of an associated field, like temperature, electric potential, or pressure. This relationship is condensed into a material-dependent parameter called the transport coefficient. For the previous examples, the associated transport coefficients would be Fourier’s thermal conductivity, electric conductivity, and viscosity, respectively. Computational calculations of transport coefficients are a key part of MD simulations, and throughout the years, different methods to compute them have been reported. Perhaps the most direct method are non-equilibrium molecular dynamics (NEMD) simulations,^6^ which rely on inducing a perturbation on the system, such as a small gradient in the associated field to the transport coefficient, to then compute the response of the system in terms of an associated current. This technique has been widely used for the study of thermal conductivity,^7–9^ viscosity,^10–12^ or electric conductivity,^13^ among others.

To avoid carrying out NEMD simulations, other popular way to compute transport coefficients is to use equilibrium MD simulations in conjunction with the Green–Kubo (GK) relations.^14,15^ These are a consequence of the fluctuation–dissipation theorem^16^ and relate the transport coefficients to fluctuations that take place in equilibrium, thus bypassing the need to introduce external perturbations into the simulations. This method is also widely used, with numerous examples found in the literature, such as the study of thermal conductivity in solids^17–19^ or of charge, particle, and momentum transport in ionic systems.^20–29^

Among the latter, ionic liquids (ILs) are a particularly interesting set of materials. These are room-temperature molten salts, frequently synthesized from a small inorganic anion and a larger organic cation, which leads to an unfavorable lattice structure and thus prevents solidification.^30,31^ In the past years, ILs have attracted a great deal of attention due to the customizability of their properties, which results from the large number of possible cation–anion pairs that can form an IL. Nevertheless, the determination of their transport properties, from MD simulations is complicated since the dynamics of these systems can be sluggish due to the strength of Coulombic interactions, thus leading to the need for long and time-consuming simulations. Moreover, the use of the GK relations is not straightforward since problems due to insufficient sampling or statistical accuracy tend to arise. Many efforts have been devoted to developing simulation procedures that minimize errors,^32^ quantifying the uncertainty and convergence requirements of the GK method^33–35^ or developing alternative schemes to obtain the transport coefficient from the microscopic currents.^36^ Moreover, in recent years, the development of new parametrizations for ILs that take into account polarization effects,^37,38^ as well as neural network potentials trained from ab initio calculations^39,40^ have severely improved the description of these materials, opening the door for faster, more realistic dynamics and a better determination of experimental properties.

In this work, we report a framework to compute the transport coefficients from the microscopic currents through the GK formalism, and it has been implemented as a lightweight Python package that enables both the calculation of the transport properties and the measurement of currents from MD simulations. In the following sections, we provide the theoretical foundation for this method, as well as a brief overview of the capabilities of the developed package. Then, the method is used to compute the transport properties of a test system, a protic IL. The behavior of the method under different conditions, as well as its performance when compared to alternative approaches found in the literature, is examined.

## Methodology

### Theoretical Background

In the Green–Kubo (GK) formalism, the components of the transport tensor γ^αβ^ are given (up to a time-independent multiplicative constant) by

1 $$\gamma^{\alpha \beta }=\int_{0}^{\infty }\left\langle J^{\alpha }\left(t\right)J^{\beta }\left(0\right)\right\rangle \;dt=\int_{0}^{\infty }C^{\alpha \beta }\left(t\right)\;dt$$

where $J^{\alpha }$ is a given component of the microscopic current associated with the transport property, and *C* ^αβ^(*t*) is called the current autocorrelation function (CAF). When processing a MD trajectory, the currents become a discrete series, and the value of the CAF at a given multiple *k* of the time step Δ*t* can be calculated as

2 $$C^{\alpha \beta }\left(k\Delta t\right)\equiv C_{k}^{\alpha \beta }=\frac{1}{N-k}\sum_{i=0}^{N-k-1}J_{i+k}^{\alpha }\cdot J_{i}^{\beta }$$

where *N* is the number of steps in the simulation, and $J_{k}^{\alpha }=J^{\alpha }\left(k\Delta t\right)$ . Frequently, instead of obtaining a correlation function with the length of the whole trajectory, the latter is divided into intervals of equal length. A correlation function $\left\langle J^{\alpha }J^{\beta }\right\rangle$ can be calculated from each interval, and the average CAF is given by

$$C_{k}^{\alpha \beta }=\frac{1}{M}\sum_{A=1}^{M}\langle J^{\alpha }\cdot J^{\beta }\rangle_{k}^{\left(A\right)}$$ 3

where the index *A* runs over the *M* different intervals. Moreover, the statistical uncertainty of the CAF is given by (a complete derivation is available in the Supporting Information)­

$$u\left(C_{k}^{\alpha \beta }\right)=\frac{\sigma_{k}^{\alpha \beta }}{\sqrt{M\left(N-k\right)}}=\frac{1}{\sqrt{M\left(N-k\right)-1}}{\left[\frac{1}{M}\overset{M}{\underset{A=1}{\sum }}{\left\langle {\left(J^{\alpha }\right)}^{2}\cdot {\left(J^{\beta }\right)}^{2}\right\rangle }_{k}^{\left(A\right)}-{\left(C_{k}^{\alpha \beta }\right)}^{2}\right]}^{1/2}$$ 4

where σ_*k*_^αβ^ is the standard deviation of the *k*th value of the CAF. It is interesting to remark that the determination of the uncertainty just involves the calculation of the correlation function for the squares of the currents, which has the same time complexity as that of the CAF, O(Nlog⁡N) using a Fast Fourier Transform method. Therefore, this quantity can be determined without exceedingly high computational costs.

To estimate the transport coefficient, γ^αβ^, we turn to the running integral of the CAF. Using a common trapezoidal scheme, the values of the running integral are given by

5 $$I_{k}^{\alpha \beta }=\frac{\Delta t}{2}\sum_{i=0}^{k}\left(C_{i}^{\alpha \beta }+C_{i+1}^{\alpha \beta }\right)$$

The uncertainty of the running integral can be calculated through standard error propagation. By neglecting the covariance between the measurements of adjacent values of the CAF, it is given by

6 $$u\left(I_{k}^{\alpha \beta }\right)=\frac{\Delta t}{2}\sqrt{\sum_{i=0}^{k}\left(u^{2}\left(C_{i}^{\alpha \beta }\right)+u^{2}\left(C_{i+1}^{\alpha \beta }\right)\right)}$$

It will be shown later that this uncertainty grows as $\sqrt{k}$ as time increases. Thus, the estimation of γ^αβ^ becomes a difficult problem since even if the running integral forms a plateau, not all points belonging to it will have the same statistical significance. This is problematic, since one of the most common methods to obtain the transport coefficient from $I^{\alpha \beta }$ is to average it over some region,^32^ which would result in neglecting these changes in the uncertainty. In turn, this would lead to a worse estimation of the transport coefficient since data points with fewer statistical accuracy would not be weighed accordingly. To account both for the fluctuations in the plateau and the different uncertainties, we define the running transport coefficient as the following weighted average

7 $$\gamma_{i}^{\alpha \beta }=\frac{\sum_{k=i}^{N}I_{k}^{\alpha \beta }/u^{2}\left(I_{k}^{\alpha \beta }\right)}{\sum_{k=i}^{N}u^{-2}\left(I_{k}^{\alpha \beta }\right)}$$

whose statistical uncertainty is given by

8 $$u\left(\gamma_{i}^{\alpha \beta }\right)=\sqrt{\frac{1}{N-i}\frac{\sum_{k=i}^{N}{\left(\gamma_{i}^{\alpha \beta }-I_{k}^{\alpha \beta }\right)}^{2}/u^{2}\left(I_{k}^{\alpha \beta }\right)}{\sum_{k=i}^{N}u^{-2}\left(I_{k}^{\alpha \beta }\right)}}$$

Finally, if the sequence γ_*i*_^αβ^ shows a plateau for a wide range of averaging origins, the value at said plateau can be identified with the transport coefficient γ^αβ^. In this way, the need for arbitrary cutoffs is eliminated from the method since once the plateau is identified, all points within it will be equivalent up to statistical uncertainty. Finally, it is usually the case that the average isotropic transport coefficients are reported rather than the individual components of the transport coefficient tensor. The running average of the isotropic transport coefficient γ_*i*_ can be calculated as

9 $$\gamma_{i}=\frac{\sum_{\alpha }\gamma_{i}^{\alpha \alpha }/u^{2}\left(\gamma_{i}^{\alpha \alpha }\right)}{\sum_{\alpha }u^{-2}\left(\gamma_{i}^{\alpha \alpha }\right)}$$

as well as its uncertainty

10 $$u\left(\gamma_{i}\right)=\sqrt{\frac{1}{2}\frac{\sum_{\alpha }{\left(\gamma_{i}^{\alpha \alpha }-\gamma_{i}\right)}^{2}/u^{2}\left(\gamma_{i}^{\alpha \alpha }\right)}{\sum_{\alpha }u^{-2}\left(\gamma_{i}^{\alpha \alpha }\right)}}$$

As in the case of the general components, the value of the transport coefficients can be taken from the plateau value of the sequence. This methodology is implemented in kute (green-Kubo Uncertainty-based Transport properties Estimator), a Python package that allows both the estimation of the transport coefficients and the calculation of the microscopic currents.

## Simulation Details

To evaluate the methodology, polarizable MD simulations of a protic IL were carried out in OpenMM, version 7.6.^41^ The chosen IL was ethylammonium nitrate (EAN), an archetypal IL that is well-known for its ability to form a hydrogen bond network. Thus, to properly capture the interactions within the system, the polarizable CL&Pol force field developed by Goloviznina and co-workers^38,42,43^ was used. This force field parametrization for EAN was previously reported by the same authors.^42,44–48^ Ten independent simulation boxes containing 500 ion pairs each were created using PACKMOL,^49,50^ and then OpenMM input files for the polarizable simulations were constructed using fftool^51^ and pol_openmm.^52^ The systems underwent energy minimization with a tolerance of 10 kJ/mol. Then, stabilization runs were carried out in the NpT ensemble for 10 ns. Following that, further stabilization runs were performed in the *NVT* ensemble for an additional 5 ns. Finally, the systems evolved in the *NVT* ensemble for 50 ns. Bonds involving hydrogen atoms were kept frozen. For all simulations, a constant time step of 1 fs was employed. During production runs, the kute.reporters module was used to save the electric current and the off-diagonal components of the pressure tensor, with recording frequencies of 1 and 5 fs, respectively. In addition to the 50 ns production runs, shorter simulations in the *NVT* ensemble were carried out for 500 ps. During these simulations, the velocities of the centers of mass of each molecule were recorded every 1 fs for the calculation of the diffusion coefficients.

During all MD simulations, temperature was held constant by means of a temperature-grouped Nosé-Hoover thermostat,^53^ implemented in the velocityverletplugin code.^54^ The translational center of mass motion was thermalized at 298.15 K with a 10 ps^–1^ collision frequency, while the temperature of Drude particles was kept at 1 K, with a 40 ps^–1^ collision frequency. In the simulations in the NpT ensemble, pressure was held at 1 bar using a Monte Carlo barostat.^55,56^ To ensure the stability of the trajectory, the maximum distance between a core and its Drude particle was set at 0.2 Å through a hard wall constraint. Smooth particle mesh Ewald electrostatics were used to account for the long-range Coulombic interactions, with a real space cutoff of 12 Å and an error tolerance of 10^–5^. Finally, van der Waals forces were truncated above the same cutoff.

## Results and Discussion

### Electric Conductivity

In the case of the electric conductivity κ, the GK theorem takes the form

11 $$\kappa^{\alpha \beta }=\frac{1}{Vk_{B}T}\int_{0}^{\infty }\left\langle J^{\alpha }\left(t\right)\cdot J^{\beta }\left(0\right)\right\rangle dt$$

where *V* is the volume, *k*_B_ is Boltzmann’s constant, *T* is the temperature, and *J* ^α^ is a component of the collective electric current of the system, which can be calculated as

12 $$J=\sum_{k}Q_{k}V_{k}$$

where the sum runs over all molecules in the system, each with total charge *Q*_*k*_ and with the center of mass velocity **V**_**k**_. The CAF was computed from the recorded electric currents by using different simulation lengths, always splitting the total time series into 1 ns chunks. The results for the *C* ^*xx*^ component of the correlation function are shown in Figure 1a. The CAF shows a series of damped oscillations and then rattles around zero until very large lag times, where the lack of sampling induces erratic behavior. The simulation length does not seem to influence the shape of the CAF, which is to be expected given that the typical correlation time is around 1 ps, well below the lower limit of 5 ns for the simulation duration. However, even though it does not affect the behavior of the CAF, simulation time does have an important effect on its uncertainty. In Figure 1b, it can be seen that the shape of the uncertainty is the same regardless of duration. For long lag times, the uncertainty becomes constant, coinciding with the region where the CAF oscillates around zero. Increasing the simulation time lowers this constant uncertainty value, as shown in Figure 1c, where the evolution of the plateau value is represented against the simulation duration. It can be seen that as simulation time increases, the accuracy of the CAF values improves as it is expected. Moreover, it can be seen that this decrease in uncertainty evolves as *t*_s_^–1/2^, with *t*_s_ being the duration of the simulation. This was shown theoretically by Zwanzig and Narinder,^33^ under the assumption that the microscopic current behaves as a random Gaussian variable. We can see that this is indeed the case, at least for sufficiently large times.

**Figure 1:**
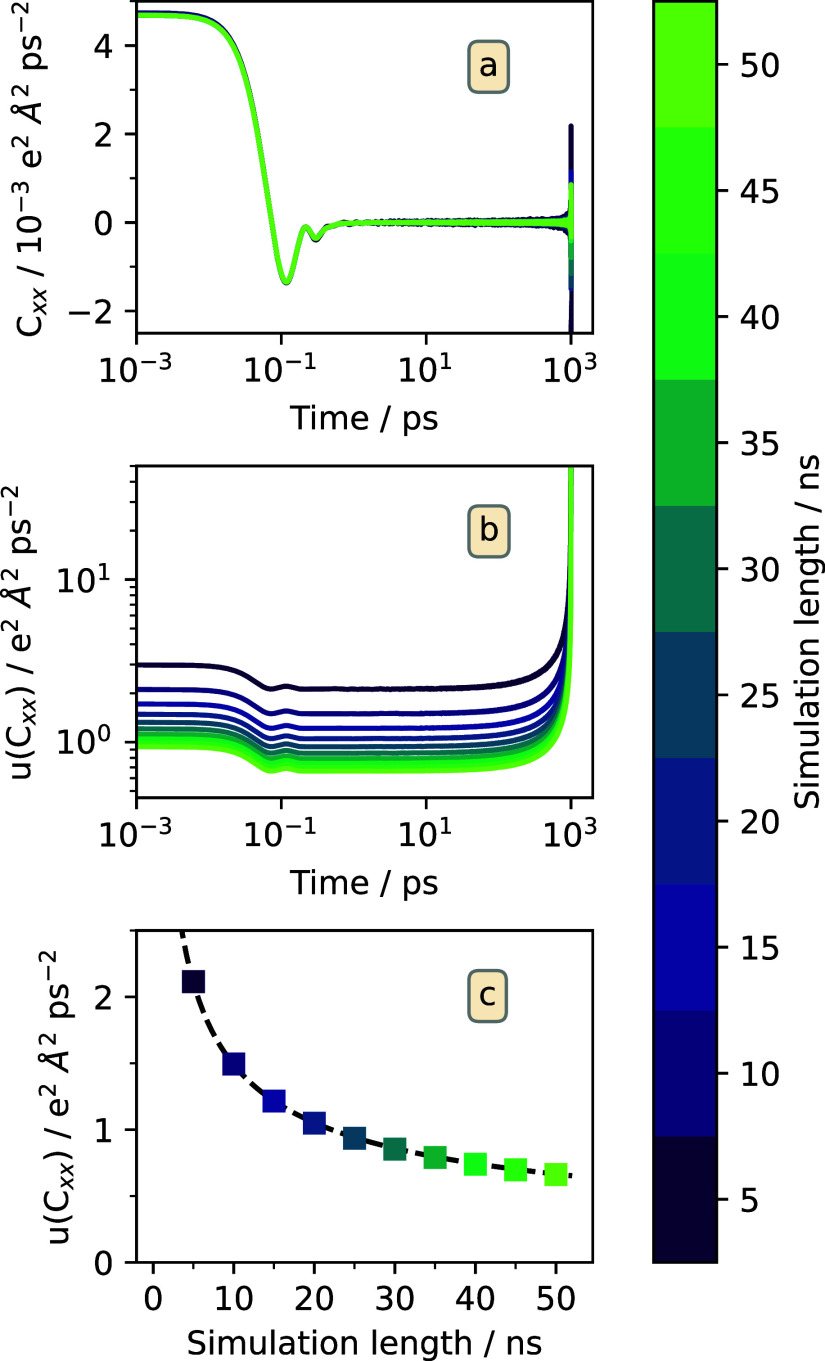
(a) Current autocorrelation functions for different simulation lengths. (b) Uncertainties of the CAFs computed according to eq 4. (c) Evolution of the plateau value of the uncertainty with simulation length. The dashed line represents a fit to a function of the form *A*·*t* ^–1/2^.

The behavior of the uncertainty observed in Figure 1b implies that, as it was said in the theoretical introduction, the points in the cumulative integral will not have the same uncertainty. From eq 6, and taking into account that *u*(*C*_*i*_^αβ^) ≃ *u*_0_ for long times, the uncertainty of the running integral will grow as

13 $$u\left(I_{k}^{\alpha \beta }\right)∼\frac{\Delta t}{2}u_{0}\sqrt{k}$$

This growing uncertainty for the cumulative integral was already observed by Oliveira et al.,^35^ who equated the behavior of the integral to a Gaussian random walk. Here, this comes as a consequence of the constant uncertainty of the CAF when it oscillates around zero.

Once the behavior of the CAF has been established, the running conductivity and its uncertainty can be calculated using eqs 7 and 8. In Figure 2, the results for each of the ten independent simulations are represented. It can be seen that even though some of the simulations show a plateau in their running conductivities, others (replica 4) do not. This is a consequence of the collective nature of the electric current and therefore of the electric conductivity, which makes it sensible to the initial configuration of the simulation. To resolve this, the weighted average between the different running conductivities was taken, and it is displayed in Figure 2 as a solid line. This average over replicas, which can be done with tools included in kute, shows a much better convergence than that of the individual simulations, displaying a plateau for a wide range of averaging origins. The value of the plateau (3.52 ± 0.18 S/m) can be taken as the value of the electric conductivity.

**Figure 2:**
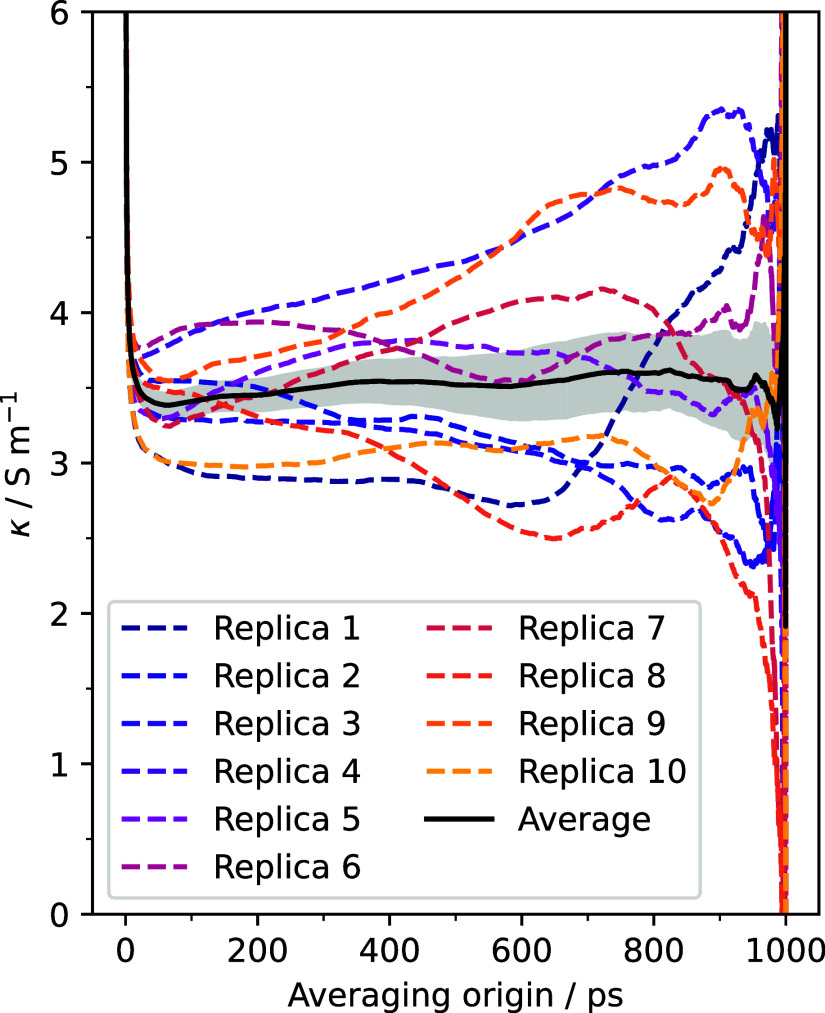
Running isotropic electric conductivity. Dashed lines represent the results for each of the ten independent simulations, while the solid line is their average. The shaded region represents the uncertainty of the average.

The previous analysis was carried out splitting the total 50 ns trajectory into intervals of 1 ns each to then compute correlation functions as averages over those intervals. This choice was driven by two key factors: maximizing statistical accuracy in the CAF by minimizing interval size and ensuring convergence of the running conductivity toward a plateau value, which requires sufficiently long CAF durations. Recognizing that both interval size and total simulation time are external parameters set by the user, it is crucial to evaluate their influence on the results. Therefore, we computed the evolution of electrical conductivity for various simulation lengths using intervals of 1 ns and 500 ps. The results are shown in Figure 3, where it can be seen that, for sufficiently long simulation times, the values of the electric conductivity converge up to statistical uncertainty. Moreover, that value is the same for both 500 ps and 1 ns correlation functions, showing that this parameter does not greatly influence the calculated transport coefficient.

**Figure 3:**
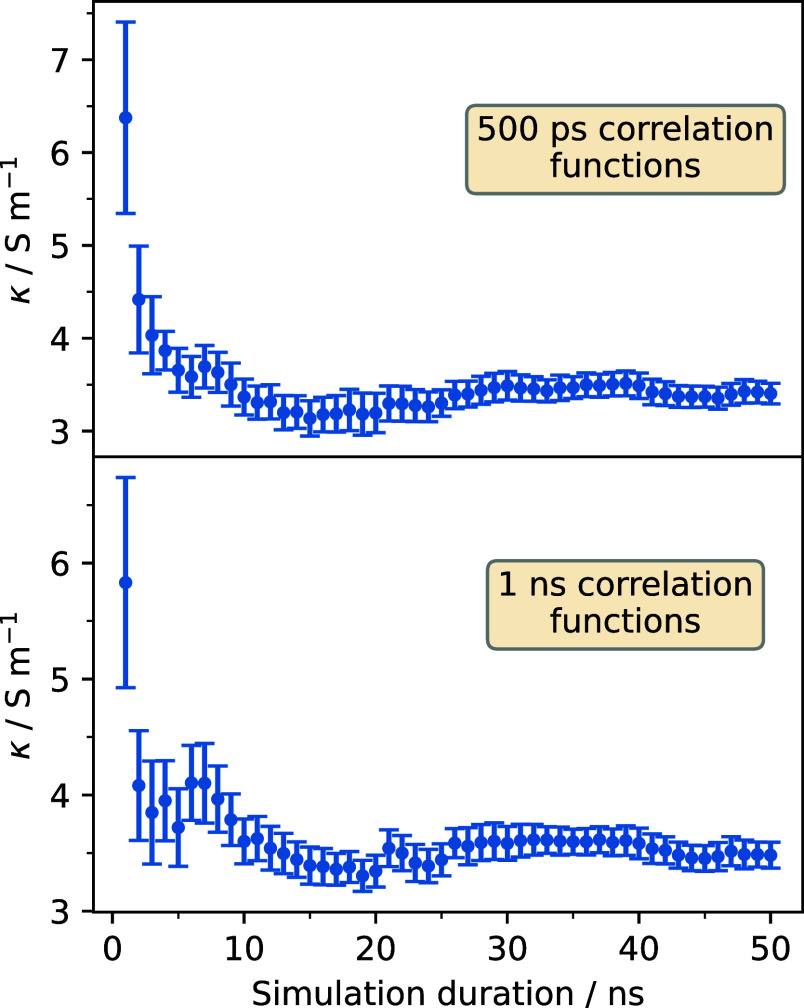
Values of the electric conductivity obtained for different simulation durations and for two different lengths of the correlation functions.

Despite the fact that kute allows for on-the-fly calculations of the electric current reduces storage requirements, saving quantities at each time step can increase simulation runtime (due to the extra computational cost of the operations and the need of communication between the simulation cores). Thus, it is interesting to see how the results change if the saving frequency for the currents decreases. This will reduce the storage space that they take up as well as the time needed to compute the trajectories, but it could introduce errors in the calculation of the integrals via the trapezoidal scheme. In Figure 4, the evolution of the calculated conductivities with said frequency for the two studied lengths of the CAF is displayed. The conductivity shows a constant value, even for a high saving frequency of 20 fs. Therefore, it is possible to decrease the recording frequency without impacting the conductivity values, although it should be stated that the point at which errors begin to appear is possibly very system dependent. Therefore, the effects of changing the saving frequency should always be tested.

**Figure 4:**
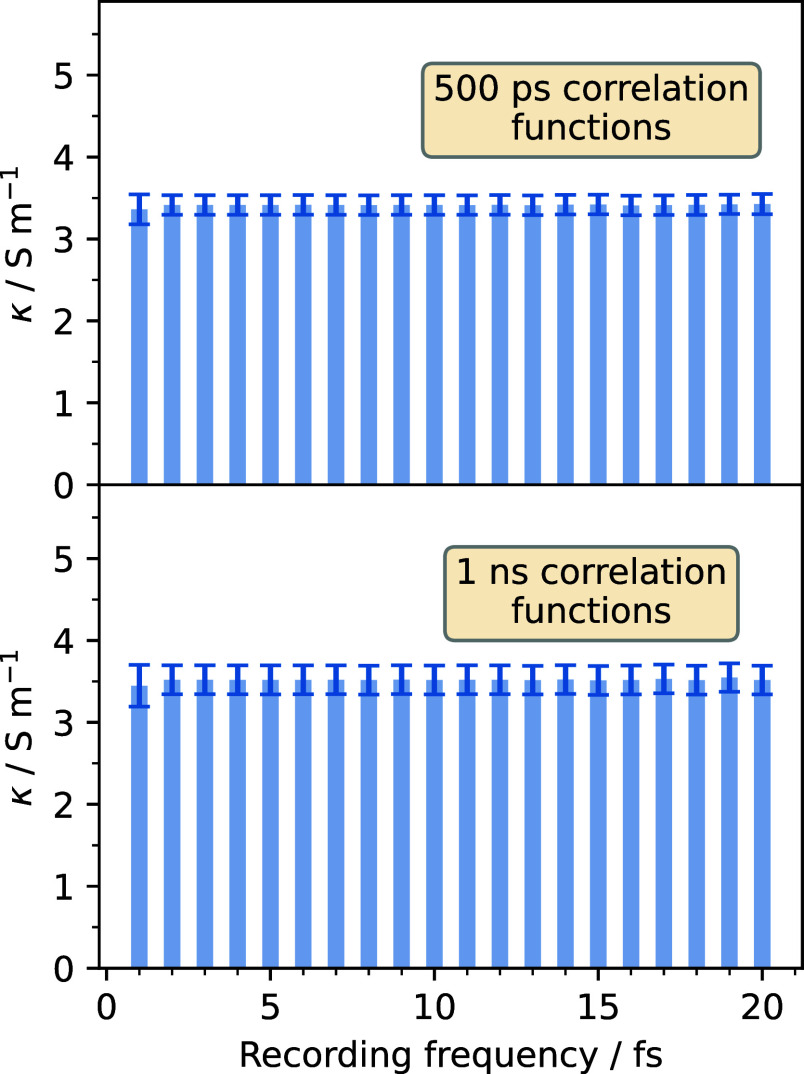
Values of the conductivity calculated for different recording frequencies and for two different lengths of the correlation functions.

Finally, it is interesting to consider the effect that taking the uncertainties into account has on the electric conductivity. If no uncertainties are computed, it is no longer possible to take averages from a given point to the end of the cumulative integral since there will be no mechanism to discard the final points where the measurements become unreliable due to poor statistics. Therefore, the averaging procedure will need to be carried out over a certain window of length *W*. Recovering the notation from the introduction, the running transport coefficient for a certain window is given by

14 $$\gamma_{i}^{\alpha \beta }\left(W\right)=\frac{\Delta t}{W}\sum_{k=i}^{W/\Delta t}I^{\alpha \beta }$$

It is important to remark that, when using this scheme, the maximum time up to which it is possible to compute the running conductivity is the length of the correlation function minus the size of the window. The calculated conductivities are shown in Figure 5 for different values of *W*. It can be seen that for small averaging windows, the results do not show a clear plateau. For larger windows, stable regions begin to appear, but the degree of stability obtained with kute is only obtained for very large values of *W*, thus losing access to a significant part of the running transport coefficient. Moreover, the fact that kute does not require specifying the value of *W* as an external parameter offers the advantage that it is not necessary to verify that results do not change under variations of this parameter.

**Figure 5:**
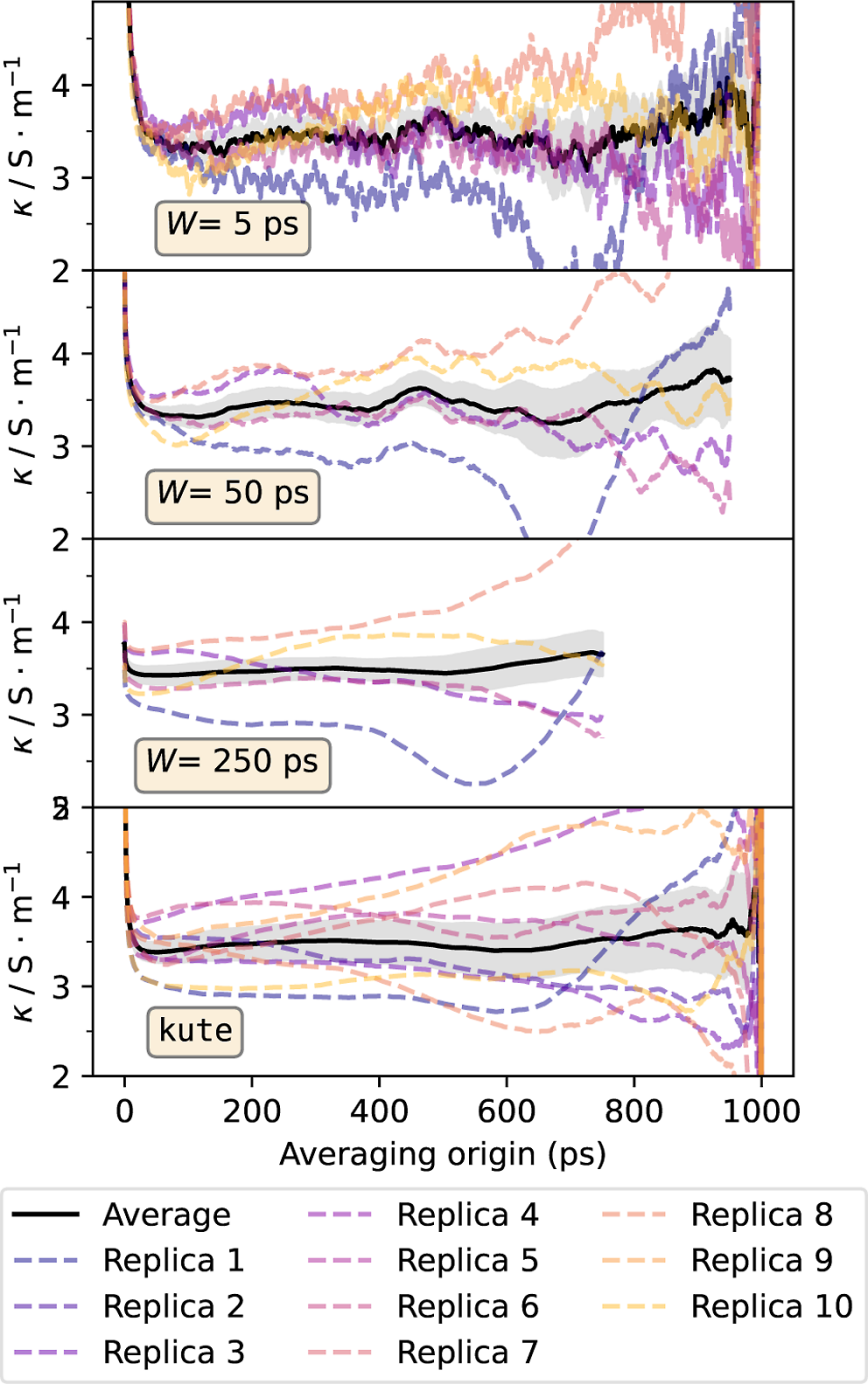
Running conductivities calculated with eq 14 for different values of the averaging window. Shaded regions indicate the uncertainty of the average.

#### Testing against Other Methods

Many methods have been reported to calculate transport properties. One of the most popular ones is to model the behavior of the CAF fitting it to certain functional forms. This can be done either fitting the whole CAF^57^ or performing the integration numerically up to some switch time, after which the decaying parts of the CAF will be fitted to a certain function, which can then be integrated analytically up to infinity to yield the transport coefficient. In this study, we focus on the latter technique. There exists a wide variety of functions in the literature that are used to describe the decay of the CAF.^58^ In this work, we study the following family

15 $$f_{k}\left(t\right)=\sum_{i=1}^{k}A_{i}e^{-t/\tau_{i}}$$

where *A*_*i*_ and τ_*i*_ are fitting parameters. The sum of exponentials is meant to represent the different relaxation processes in the system, each one with its characteristic time scale. To arrive at the values of the parameters, the decaying end of the CAF obtained for each replica was fitted to eq 15 for different values of *k*. The fitting region was chosen to maximize the value of the *R* ^2^ statistic.^58^ The resulting fits can be seen in Figures S2 and S3 of the Supporting Information. Then, a value of the electric conductivity is calculated for each replica, and a final value is obtained by averaging over the replicas. It is worthwhile to note that the *ansatz* in eq 15 is just one of a variety of possible choices, and a refinement of this assumption can lead to a better representation of the correlation function. However, there is no clear method to choose between fitting functions, and thus, the fact that the direct integration method does not require guessing this function can be seen as an advantage.

Another way to arrive at the transport coefficient is to use a mathematical equivalent to the GK theorem. These are the so-called Einstein relations,^32^ which relate the transport coefficient to the slope of the infinite time limit of the mean square displacement (MSD) of some quantity. Einstein relations rely on the same assumptions as the GK theorem and thus should yield the same result independently of the quality of the force field. Therefore, they are a useful tool to gauge whether or not kute performs a proper estimate of the GK integrals. In the case of electric conductivity, the Einstein relation takes the following form

16 $$\kappa =\frac{1}{6Vk_{B}T}\lim_{t\to \infty }\frac{d\left\langle {\left(M\left(t\right)-M\left(0\right)\right)}^{2}\right\rangle }{dt}$$

where **M** is the translational component of the dipole moment of the system, given by

17 $$M=\sum_{k}Q_{k}R_{k}$$

the sum running over all molecules in the system, with total charge *Q* and center of mass coordinates **R**. This approach is mathematically equivalent to the GK relations, and it is widely used for the calculation of conductivity in ILs.^59–61^ It should be noted that the dipole moment **M** is a collective variable of the entire system, in the same way as the electric current **J**. Therefore, its computation suffers from problems due to poor statistics, requiring multiple simulations with lengths comparable to the ones carried out in this work.^62^ To obtain the electric conductivity from the Einstein relation, an average dipole MSD was calculated by averaging the MSDs of each individual replica. The average MSD was fitted to a linear function in the interval from 3 to 3.5 ns to estimate the slope.

The calculated values obtained through these different methods are listed in Table 1. Focusing on the results obtained from the fits, it is evident that the fit to *f*_1_ does not correctly capture the transport coefficient, which indicates the existence of more than one relaxation process present in the system. The fits to *f*_2_ and *f*_3_ show results more similar to the other methods, but it should be noted that their uncertainty is 1 order of magnitude greater than that of kute. Remarkably, the results obtained with kute and through the Einstein relation are compatible with one another, which confirms that kute indeed yields a proper estimate of the GK integrals. Finally, the results can be compared to the experimental value for this quantity, which was reported as (2.26 ± 0.23) S/m by Mariani et al.^63^ The calculated values are in good agreement with experiment, at least qualitatively, due to the improvements in the dynamics provided by the polarizable force field. It should be said, however, that the ability to reproduce this number is not a matter of the method used to calculate it from MD simulations but rather of the specific parametrization of the force field. The CL&Pol force field was developed to obtain good qualitative results for the dynamic properties of ILs, but it is not intended to precisely reproduce experimental values. If a precise agreement between experiment and the values obtained either with kute or the Einstein relations was desired, it would be necessary to fine-tune the parametrization of the force field.

**Table 1:** Values of the Electric Conductivity Obtained through Different Methods

method		κ/S·m^–1^
kute		3.52 ± 0.18
Einstein relation		3.27 ± 0.30
fitting	*f*_1_	19.998 ± 0.048
	*f*_2_	5.7 ± 2.0
	*f*_3_	3.8 ± 3.0

### Viscosity

The viscosity η is also a collective property, likewise, to the electric conductivity. The GK theorem relates it to correlation functions of the pressure tensor

18 $$\eta =\frac{V}{3k_{B}T}\sum_{i\neq j}\int_{0}^{\infty }\left\langle P^{ij}\left(t\right)\cdot P^{ij}\left(0\right)\right\rangle dt$$

where *P* ^*ij*^ are the different components of the pressure tensor, although it should be noted that only the self-correlations of the off diagonal components are needed to calculate viscosity. The pressure tensor itself is given by

19 $$P=\frac{1}{V}\sum_{i}m_{i}v_{i}⊗v_{i}+\frac{1}{V}\sum_{i}r_{i}⊗f_{i}$$

where the sum runs over all atoms in the system, each one with coordinates **r**, velocity **v**, and experimenting a force **f**. In Figure 6, the running viscosity is represented for two different lengths of the correlation functions. Interestingly, and contrary to what happened in the case of electric conductivity, when using intervals of 1 ns to compute the CAF, the running viscosity does not reach a clear plateau value, and instead it is necessary to use intervals of 2 ns for it to develop. This hints at the existence of slow relaxation processes with large characteristic times, which require longer correlation functions to be properly captured.

**Figure 6:**
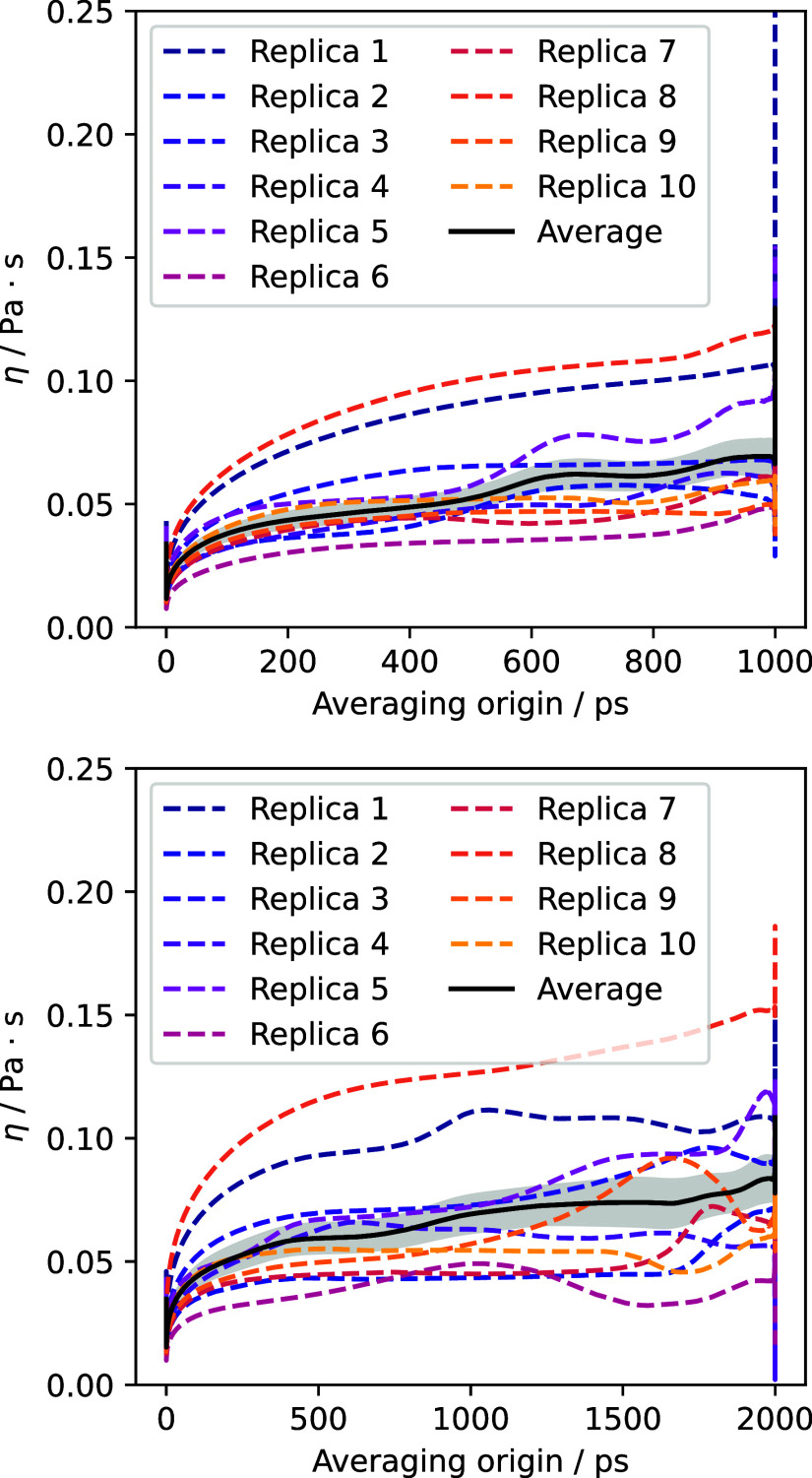
Running viscosities for each individual replica (dashed lines) and for their average (solid). The shaded area represents the uncertainty of the average. The results are shown for correlation functions with durations of 1 ns (top) and 2 ns (bottom).

When it comes to comparison against other methods, the fitting procedure described in the previous section is also widely used in the case of viscosity. For the system and parametrization studied in this work, the calculations have been already carried out,^42^ and the result is shown in Table 2 along with the value calculated in this work. It is important to note that the model function chosen by the authors was a slightly different one, which they found to be more accurate to reproduce the decaying behavior. The function is given by

20 $$f\left(t\right)=a\cdot \exp\left(-t^{\alpha }\right)$$

where *a* and α are fitting parameters. It can be seen that, as was the case with electric conductivity, kute offers similar values as the fitting approach while yielding a lower uncertainty. In this case, however, the agreement with experiment is not as good as in the case of electric conductivity since according to Mariani et al., the viscosity is reported to have a value of (33.9 ± 1.1 mPa·s).^63^ Again, the polarizable force field yields a value in the correct order of magnitude, but in order to exactly replicate experiment, more effort would need to be devoted to improving the parametrization.

**Table 2:** Values of the Viscosity Obtained through Different Methods

method	η/mPa·s
Kute	74.0 ± 9.5
fitting	64 ± 20

### Diffusion Coefficients

The diffusion tensor for a certain chemical species A is related through the GK theorem to integrals of the velocity autocorrelation function (VACF)

21 $$D_{S}^{\alpha \beta }=\frac{1}{N_{A}}\sum_{i=1}^{N_{A}}\int_{0}^{\infty }\left\langle V^{\alpha }\left(t\right)\cdot V^{\beta }\left(0\right)\right\rangle dt$$

where the sum runs over the *N*_A_ molecules of species *A*, each with a center of mass velocity **V**. In contrast with the electric conductivity and viscosity, the diffusion coefficients are single-particle properties. That is, for each trajectory, *N*_A_ correlation functions can be calculated and averaged to yield the total VACF, which greatly increases statistics. Because of that, the short 500 ps simulations performed in this work are sufficient to achieve convergence. Each one was split into two intervals of 250 ps for the computation of the VACF, and thus, the number of intervals for the computation is 2*N*_S_, which is equal to 1000 for both cations and anions. The results can be seen in Figure 7, where plateaus in the average running diffusion coefficients are observed in the region between 150 and 200 ps for both cations and anions. Remarkably, the behavior of each individual replica is much more similar to that of the average than that in the case of electric conductivity and viscosity. This is due to the aforementioned fact that diffusion coefficients are single particle properties, and thus, for each replica, they are calculated by averaging over multiple particle trajectories, in contrast with collective variables for which only one series of values for the current is available for each replica.

**Figure 7:**
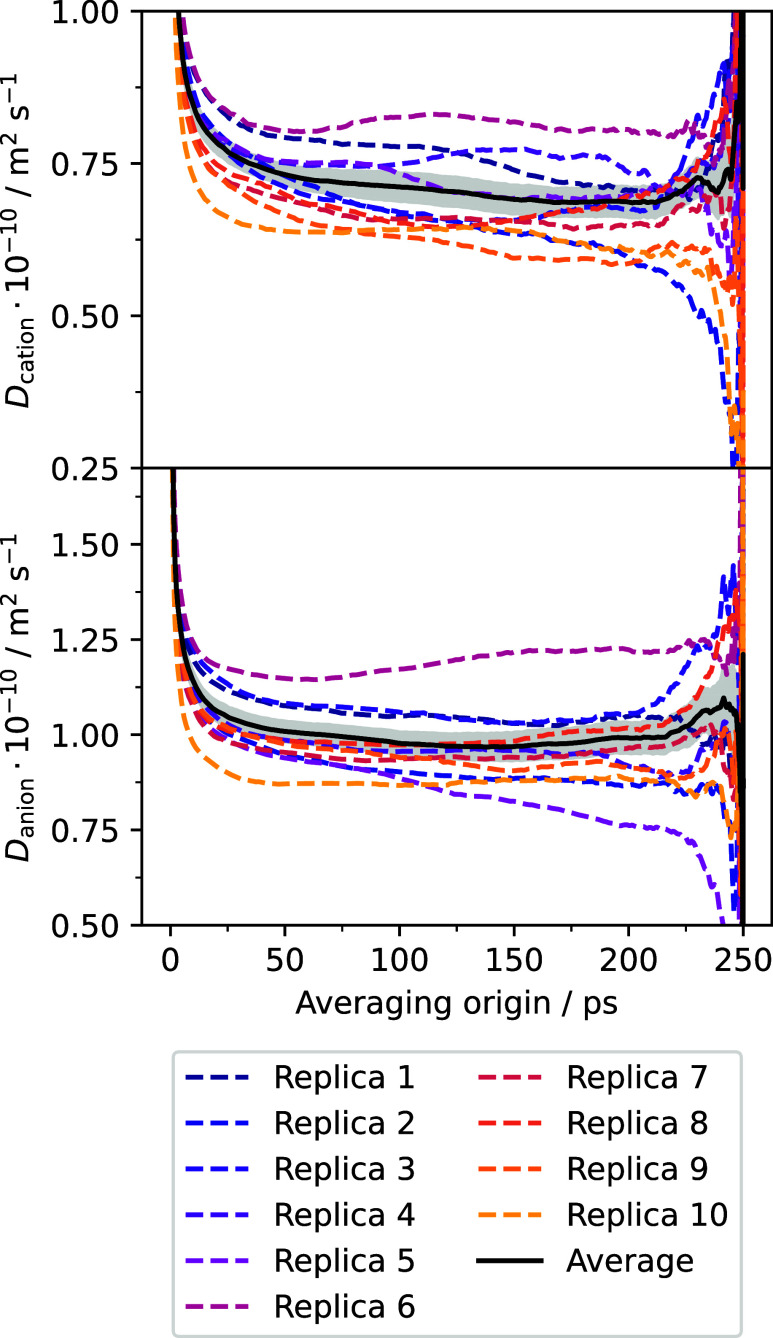
Running diffusion coefficient for cations (top) and anions (bottom). Dashed lines represent the results for each individual replica, while the solid line is the average over replicas. Shaded regions indicate the uncertainty of the average.

To compare the results with different methods, both the fitting approach and Einstein relations can be used. For this property, the latter takes the form

22 $$D_{S}=\frac{1}{6}\lim_{t\to \infty }\frac{d\left\langle {\left(R\left(t\right)-R\left(0\right)\right)}^{2}\right\rangle }{dt}$$

where **R** is the position of the center of mass of molecules of species *S*. Moreover, the average is also performed over all particles of the same species. Diffusion coefficients were computed from the Einstein relation in a manner analogous to the calculation of electric conductivity using the 50 ns runs. The fitting of the MSD was performed between 10 and 10.5 ns.

The values obtained with kute, the Einstein relation, and the fitting approach are shown in Table 3. It can be seen that a very good agreement is obtained between the Einstein relation and the results obtained with kute. This suggests an important advantage of the GK method against the Einstein formalism since even though the simulations required for the calculation of the integrals and correlation functions are much shorter than the large 50 ns runs, the obtained results are compatible with each other. Regarding the values obtained through the fitting method, they are 1 order of magnitude greater than the others. In light of the experimental values^64^ of (4.570 ± 0.002) × 10^–11^ and (5.65 ± 0.04) × 10^–11^ m^2^·s^–1^ for the cation and the anion, respectively, this indicates the lack of accuracy of the fitting method since it does not correctly predict the order of magnitude. It could be the case that a refinement of the fitting function could result in a better prediction, but if that was the case, the fact that the results can vary so much with changes to the model function could also be seen as a liability of the method, which is not exhibited by kute or Einstein relations.

**Table 3:** Values of the Diffusion Coefficients Obtained through Different Methods

method		*D*_cation_/10^–11^ m^2^·s^–1^	*D*_anion_/10^–11^ m^2^·s^–1^
kute		6.86 ± 0.25	9.92 ± 0.41
Einstein relation		6.82 ± 0.13	9.43 ± 0.15
fitting	*f*_1_	39.0 ± 2.3	30.9 ± 1.2
	*f*_2_	19.61 ± 0.57	25.89 ± 0.73
	*f*_3_	18.98 ± 0.51	18.7 ± 1.2

Finally, it is worthwhile to mention that if diffusion coefficients are available, then it is possible to use them to estimate electric conductivity. If interionic correlations are negligible,^65^ eq 11 reduces to the so-called Nernst–Einstein (NE) equation

$$\kappa_{NE}=\frac{e^{2}}{Vk_{B}T}\underset{i}{\sum }N_{i}\left|z_{i}^{2}\right|D_{i}$$ 23

where the sum runs over the different chemical species in the system, each with valence *z*, while *N* represents the number of species of each kind present in the system. This relation only holds for very dilute ionic systems but due to large and not understood compensations between ionic correlations,^66^ it has proven to sometimes be accurate in the determination of conductivity in ILs, and its widely used in the literature.^67,68^ To obtain a better estimate, the Yeh-Hummer correction^69^ is usually applied to account for finite size effects

24 $$D_{A}^{YH}=D_{A}+\frac{2.8373k_{B}T}{6\pi \eta L}$$

where *L* is the length of the simulation box. For the computed values of viscosity, the correction of the diffusion coefficients is under 1%. Applying the correction, the value of the NE conductivity is (7.06 ± 0.20) S/m, which, despite being much higher than the one computed from the GK theorem or through the Einstein relations, is of the same order of magnitude.

## Conclusions

In this contribution, we introduced kute, an algorithm that can be used to compute transport coefficients using the GK method and taking into consideration the statistical uncertainty of the different points of the correlation functions. To validate the methodology, polarizable MD simulations of the protic IL EAN were carried out, and kute was used to compute the electric current, components of the pressure tensor, and center of mass velocities, which in turn were used to compute electric conductivity, viscosity, and diffusion coefficients. The results were compared against traditional methods to estimate the GK integrals, and it was found that kute yields more accurate estimates. Moreover, the transport coefficients were also calculated using Einstein relations, and the obtained results were compatible with kute up to statistical uncertainty. Additionally, it was shown that the results obtained with kute are independent of several user-specified values, such as the length of the simulation, the recording frequency for the microscopic currents, and the length of the correlation functions.

This new methodology for the calculation of the GK integrals offers several advantages compared to the common methodologies available in the literature. When compared with methods such as direct integration or fittings to the correlation functions, kute eliminates the need for arbitrary cutoffs since it only relies on the identification of a plateau in the running transport coefficient. Moreover, it was found that in the case of single particle properties, kute achieves the same level of accuracy with short simulations than the Einstein relations do using trajectories that are larger by an order of magnitude. This results in a significant improvement in the speed at which these properties can be calculated from simulations, which can be a great advantage for the screening or characterization of a large number of compounds.

Finally, the algorithm has been implemented in a lightweight python package. The software allows both for the calculation of transport coefficients for a given microscopic current and for the calculation of said currents from MD trajectories. It is compatible with many popular trajectory and topology formats and with widely used MD suites such as GROMACS, LAMMPS, and OpenMM, in some cases allowing for on-the-fly calculations of the microscopic currents, greatly reducing data storage requirements. The kute package is available for free in a public repository as well as on the Python Package Index.

## Supplementary Material



## Data Availability

MD simulations were performed in OpenMM (https://openmm.org/), version 7.6 ^41^ in conjunction with the temperature-grouped Nosé-Hoover thermostat plugin available at https://github.com/z-gong/openmm-velocityVerlet.^54^ Initial configurations for the simulations were created with PACKMOL (https://m3g.github.io/packmol/),^49^ whereas topology files for OpenMM were generated with the fftool (https://github.com/paduagroup/fftool)^51^ and pol_openmm (https://github.com/paduagroup/pol_openmm)^52^ repositories. Figures in the manuscript were drawn using the Matplotlib package (https://matplotlib.org/).^70^ The kute package, entirely written in Python (https://www.python.org/),^71^ is available in the Python Package Index (https://pypi.org/project/kute).^72^ Software makes use of the numpy (https://numpy.org/),^73^ scipy (https://scipy.org/),^74^ h5py (https://www.h5py.org/),^75^ and MDAnalysis (https://www.mdanalysis.org/)^76,77^ libraries. Input files and analysis scripts are available at https://gitlab.com/nafomat/kute/-/tree/kute_paper

## Notes

Derivation of equation and additional details of the correlation and transport coefficient calculations (PDF)
