# Supplementary material for: KUTE: Green–Kubo Uncertainty-Based Transport Coefficient Estimator
Source: J Chem Inf Model. 2025 Mar 19;65(7):3477–87. doi: 10.1021/acs.jcim.4c02219 (PMC12124720; doi:10.1021/acs.jcim.4c02219)
Supplement: Supplementary file 1 [file ci4c02219_si_001.pdf]

Supporting Information: KUTE: G reen-Kubo Uncertainty-based Transport coefficient Estimator

Martín Otero-Lema,[†,‡] Raúl Lois-Cuns,[†,‡] M. A. Boado,[†,‡] Hadrián Montes-Campos,[*,†,‡] Trinidad Méndez-Morales,[*,†,‡] and Luis M. Varela[†,‡]

†*Grupo de Nanomateriais, Fotónica e Materia Branda, Departamento de Física de Partículas, Universidade de Santiago de Compostela, Campus Vida s/n, E-15782, Santiago de Compostela, Spain*

‡*Instituto de Materiais (iMATUS), Universidade de Santiago de Compostela, Avenida do Mestre Mateo 25, E-15782, Santiago de Compostela, Spain*

E-mail: hadrian.montes@usc.es; trinidad.mendez@usc.es

Derivation of Eq. 4 in the main text

Using the notation from the main text, and temporarily dropping the α and β indices for clarity, the average correlation function is given by

$$C_k = \frac{1}{M}\sum_{A=1}^{M}\frac{1}{N-k}\sum_{i=0}^{N-k-1}\mathcal{J}_i^{(A)}\mathcal{J}_{i+k}^{(A)} = \frac{1}{M}\sum_{A=1}^{M}\langle \mathcal{J}\cdot\mathcal{J}\rangle_k^{(A)}\,, \tag{1}$$

where the index A runs over the M intervals into which the current was split. The standard deviation of the correlation can be calculated straightforwardly

$$\begin{aligned}
\sigma_k^2 &= \frac{1}{M(N-k)-1}\sum_{A=1}^{M}\sum_{i=0}^{N-k-1}\left(C_k - \mathcal{J}_i^{(A)}\mathcal{J}_{i+k}^{(A)}\right)^2 = \\
&= \frac{1}{M(N-k)-1}\sum_{A=1}^{M}\sum_{i=0}^{N-k-1}\left(C_k^2 - 2C_k\mathcal{J}_i^{(A)}\mathcal{J}_{i+k}^{(A)} + (\mathcal{J}_i^{(A)}\mathcal{J}_{i+k}^{(A)})^2\right) = \\
&= \frac{1}{M(N-k)-1}\left[M(N-k)C_k^2 - 2M(N-k)C_k^2 + (N-k)\sum_{A=1}^{M}\left\langle \mathcal{J}^2\cdot\mathcal{J}^2\right\rangle_k^{(A)}\right] = \\
&= \frac{N-k}{M(N-k)-1}\left[\sum_{A=1}^{M}\left\langle \mathcal{J}^2\cdot\mathcal{J}^2\right\rangle_k^{(A)} - MC_k^2\right] = \\
&= \frac{M(N-k)}{M(N-k)-1}\left[\frac{1}{M}\sum_{A=1}^{M}\left\langle \mathcal{J}^2\cdot\mathcal{J}^2\right\rangle_k^{(A)} - C_k^2\right],
\end{aligned} \tag{2}$$

and so the uncertainty is given by

$$u(C_k) = \frac{\sigma_k}{\sqrt{M(N-k)}} = \frac{1}{\sqrt{M(N-k)-1}}\left[\frac{1}{M}\sum_{A=1}^{M}\left\langle \mathcal{J}^2\cdot\mathcal{J}^2\right\rangle_k^{(A)} - C_k^2\right]^{1/2}, \tag{3}$$

so the covariance matrix elements are

$$u(C_k^{\alpha\beta}) = \frac{\sigma_k^{\alpha\beta}}{\sqrt{M(N-k)}} = \frac{1}{\sqrt{M(N-k)-1}}\left[\frac{1}{M}\sum_{A=1}^{M}\left\langle (\mathcal{J}^\alpha)^2\cdot(\mathcal{J}^\beta)^2\right\rangle_k^{(A)} - (C_k^{\alpha\beta})^2\right]^{1/2}. \tag{4}$$

Oscillations at the tails of correlation functions

In order to showcase the oscillating noisy behaviour of the correlation functions, in Fig. S1 part of the tail of the current autocorrelation function is shown. It can be seen that , in this regime, and due to the finite sampling of the trajectory, the relative uncertainty is much larger when compared to its value for shorter times. Moreover, it is important to notice that, even though the signal is noisy, its oscillations take place in the scales of 0.1-1 ps, while the correlation function is sampled with a frequency of 1 fs. Thus, the oscillations are correctly captured, and the trapezoidal scheme for integration is a good aproximation.

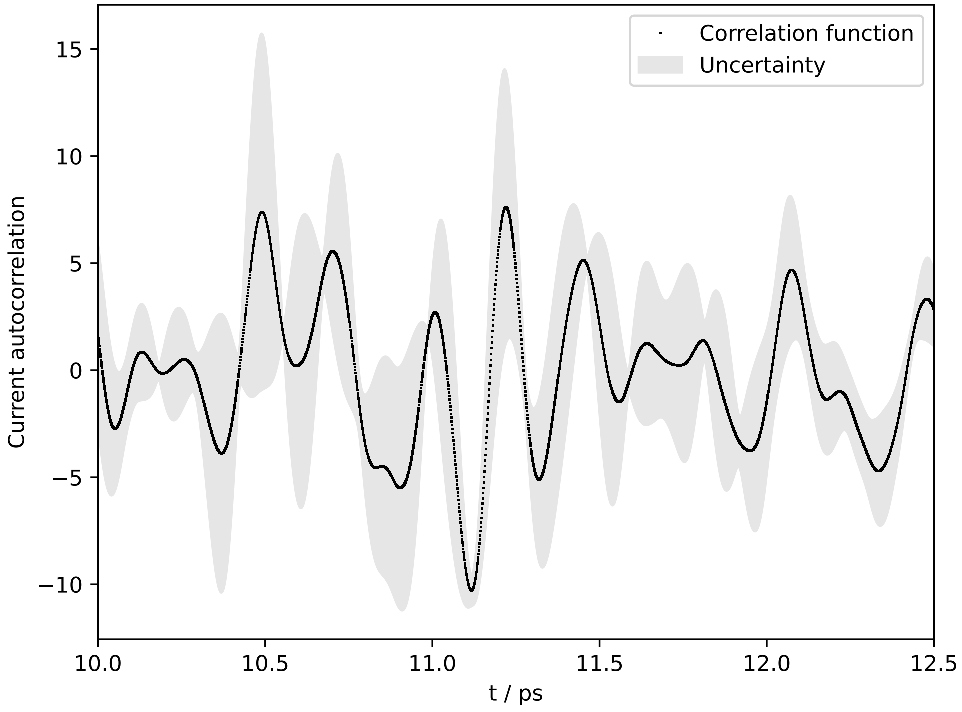

Figure S1: Oscillating part of the current autocorrelation function for one of the simulations. The shaded regions indicate the uncertainty.

Fits to the correlation functions

Here, we represent the different fits to the decaying end of the different correlation functions to the different *ansatzs* presented in the main text. In Fig. S2, the different fits to the

current autocorrelation function for one of the replicas are shown. It can be seen that, while the fit to f_1 fails to correctly capture the nature of the function, the combination of different exponentials represented by f_2 and f_3 does indeed correctly represent the general trend of the behaviour of the function. Nevertheless, it can be noticed that the true function presents several oscillations that are not correctly represented by the fits, and thus their contributions to the integral will not be accurate, unlike direct numerical integration. The case for the diffusion coefficients is analogous, as shown in Fig. S3, with the functions f_2 and f_3 closely approximating the decaying behaviour of the functions.

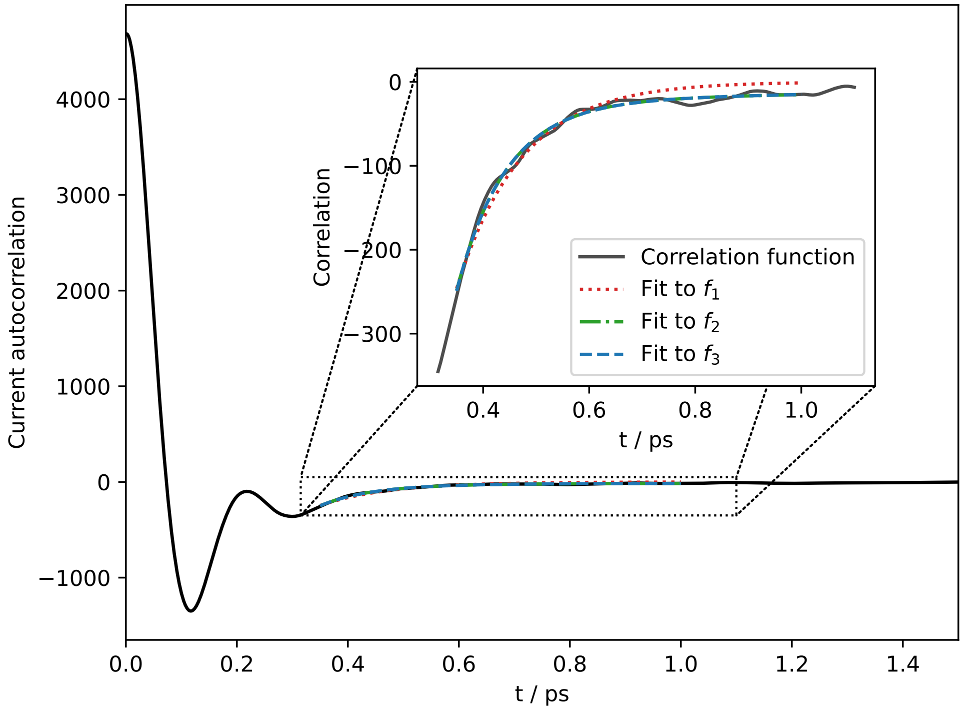

Figure S2: Current autocorrelation function, and the different fits to the *ansatzs* presented in the main text.

Details on the Einstein method

As stated in the main text, both the electic conductivity κ and the diffusion coefficients D_i can be calculated, using Einstein relations, as derivatives of mean squared displacements

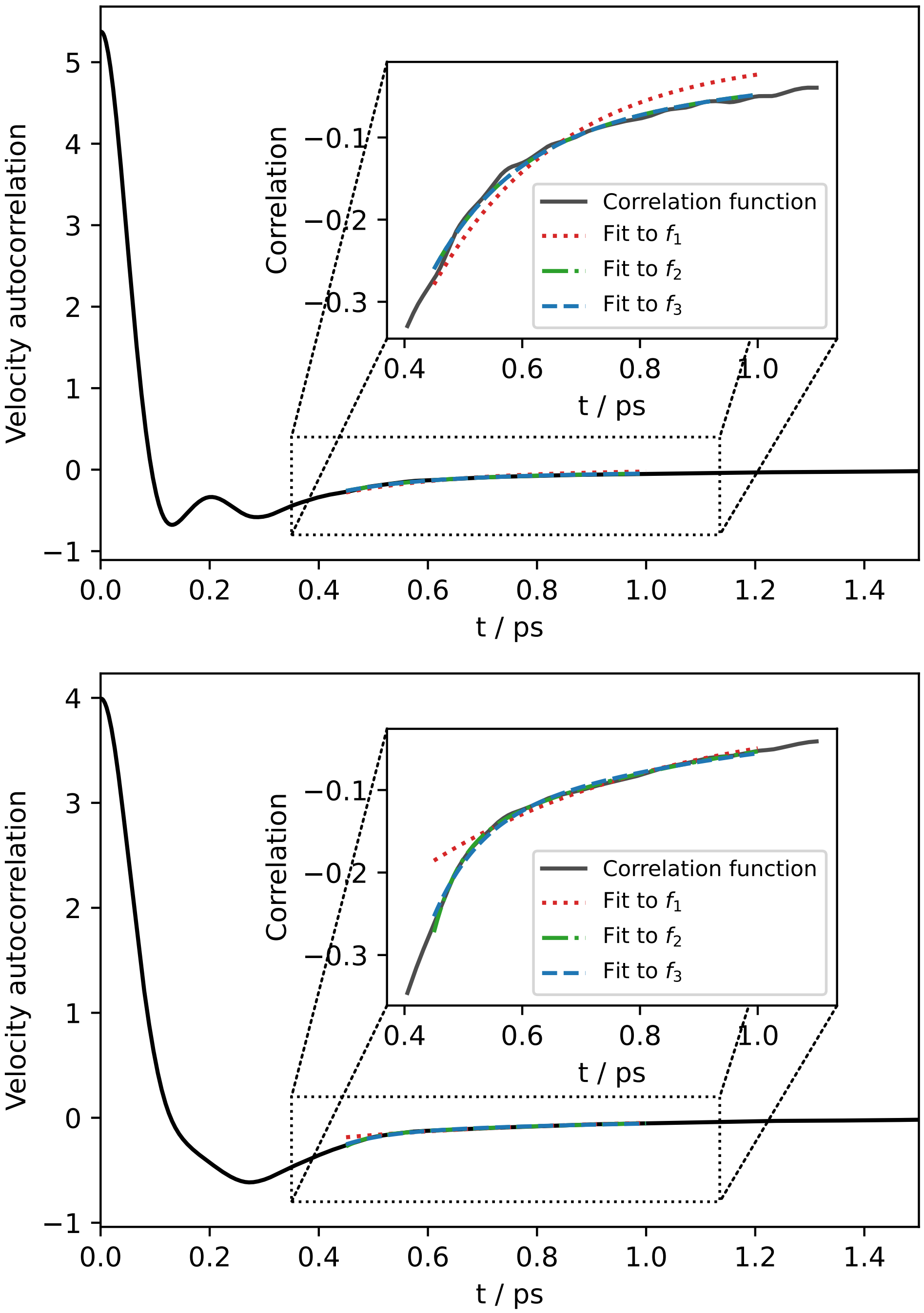

Figure S3: Velocity autocorrelation functions for the cation (top) and the anion (bottom), and the different fits to the *ansatzs* presented in the main text.

(MSDs)

$$\kappa = \frac{1}{6Vk_BT} \lim_{t \to \infty} \frac{d\langle (\mathbf{M}(t) - \mathbf{M}(0))^2 \rangle}{dt}, \tag{5}$$

$$D_i = \frac{1}{6} \lim_{t \to \infty} \frac{d\langle (\mathbf{R_i}(t) - \mathbf{R_i}(0))^2 \rangle}{dt}, \tag{6}$$

where **M** stands for the collective dipole moment of the system. In order to obtain the transport coefficient, the appropriate MSD was fitted to a linear function to extract the slope. In Figs. S4-S6 we represent both the corresponding MSD and the running transport coefficients, which were calculated using a sliding window with a width of 500 ps. The values of the transport coefficients were taken from the plateaus of these running slopes. Likewise to the procedure carried out for the Green-Kubo method, a running transport coefficient was obtained from each replica, and the final result is an average over the replicas, which becomes stable for a certain range of lag times.

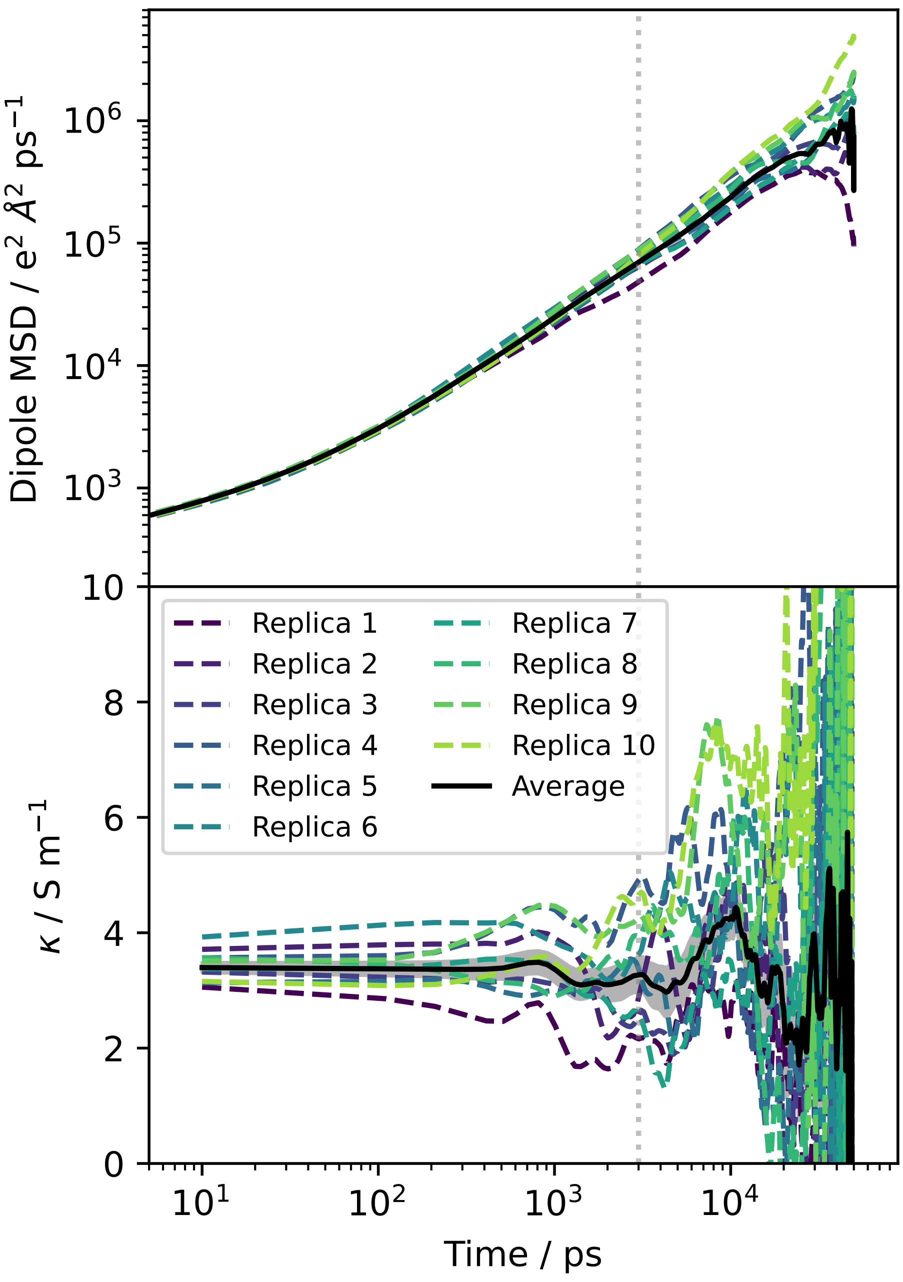

Figure S4: Dipole MSD (top) and running electric conductivity calculated using the Einstein relation (bottom). The shaded area indicates the uncertainty, while the dotted line marks the point chosen to represent the transport coefficient.

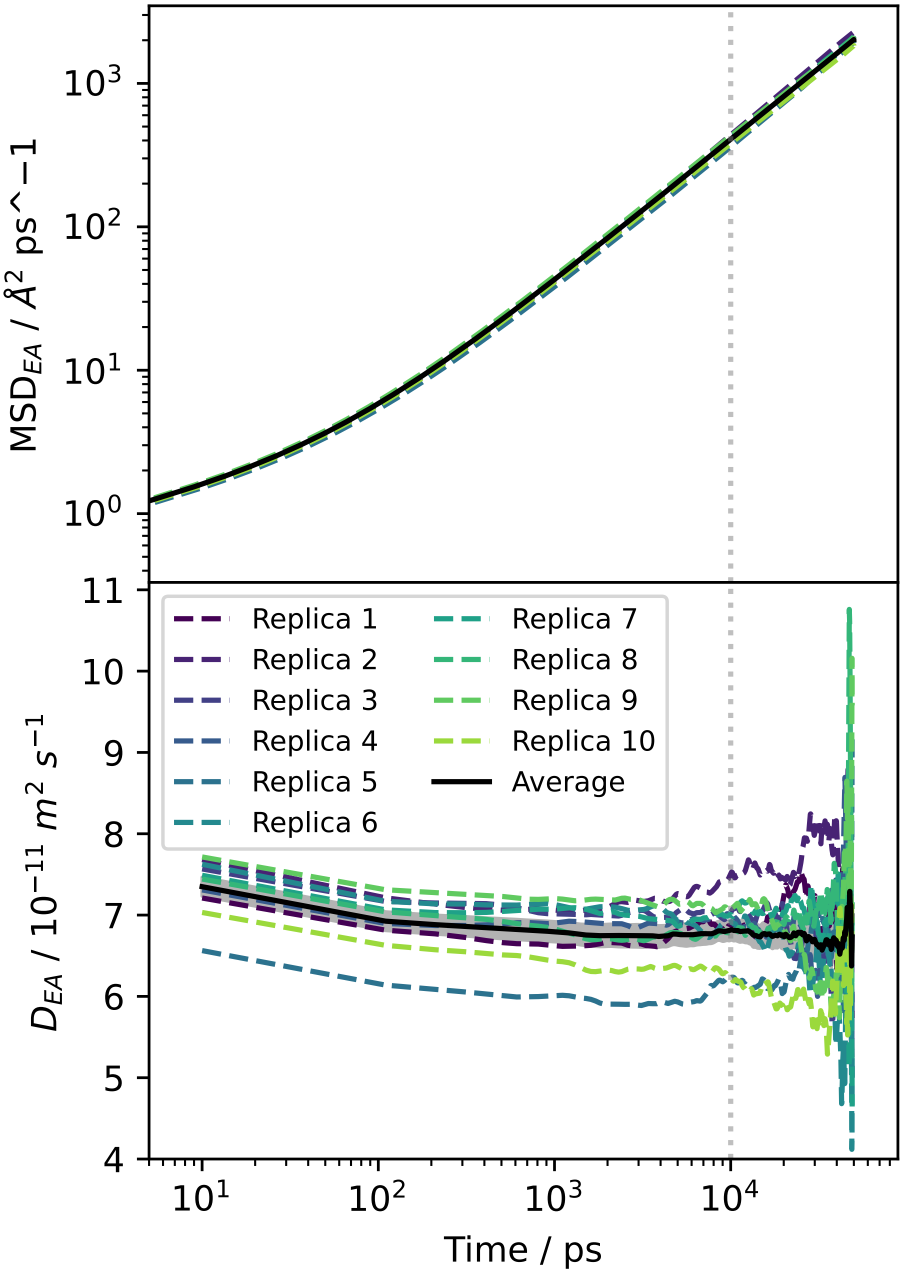

Figure S5: MSD (top) and running diffusion coefficient for the EA^+ cation. The shaded area indicates the uncertainty, while the dotted line marks the point chosen to represent the transport coefficient.

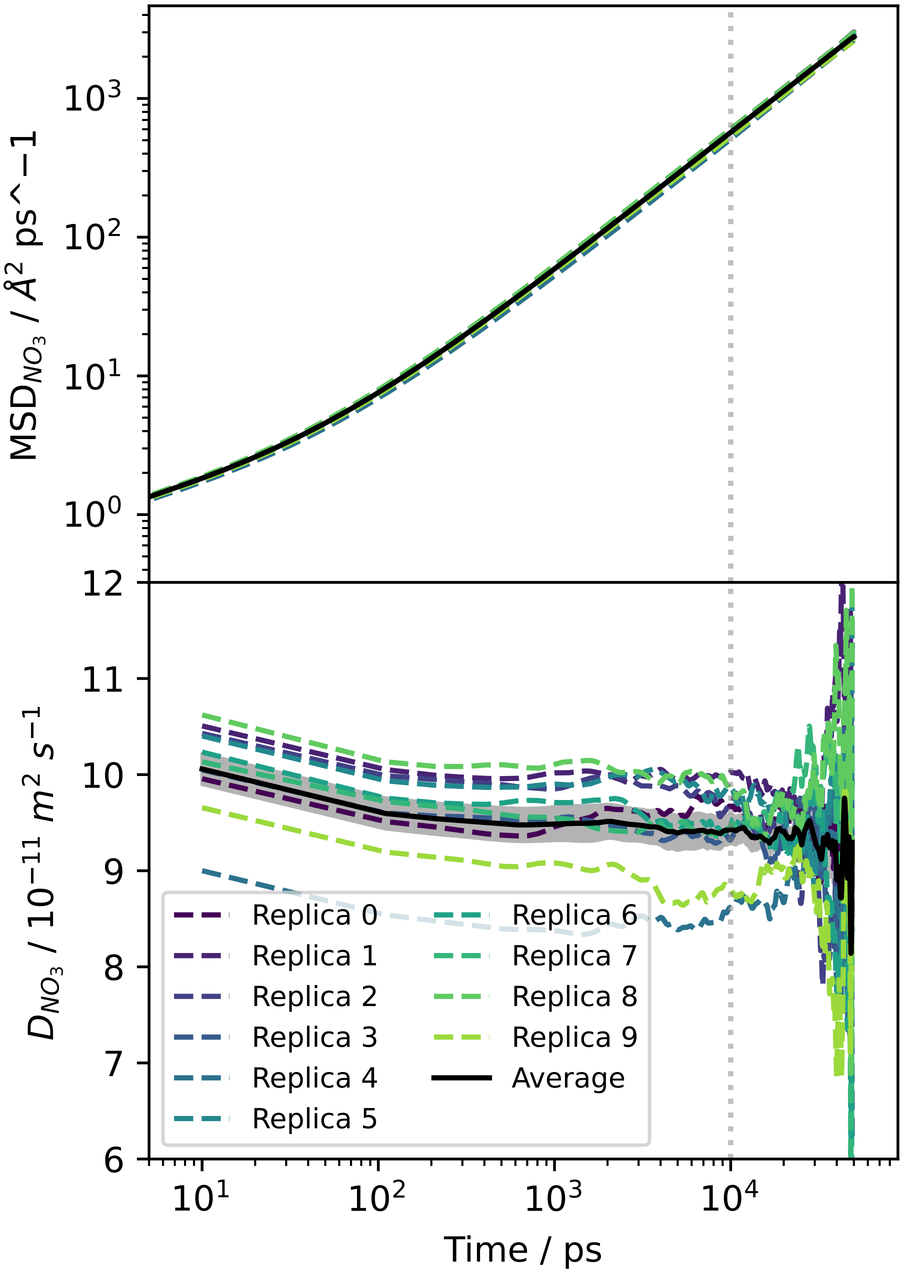

Figure S6: MSD (top) and running diffusion coefficient for the NO_3^- anion. The shaded area indicates the uncertainty, while the dotted line marks the point chosen to represent the transport coefficient.
